# Structure of the GAT domain of the endosomal adapter protein Tom1

**DOI:** 10.1016/j.dib.2016.02.042

**Published:** 2016-02-24

**Authors:** Shuyan Xiao, Jeffrey F. Ellena, Geoffrey S. Armstrong, Daniel G.S. Capelluto

**Affiliations:** a Protein Signaling Domains Laboratory, Department of Biological Sciences, Biocomplexity Institute, Virginia Tech, Blacksburg, VA 24061, USA; b Department of Chemistry and Biochemistry, University of Virginia, Charlottesville, VA 22904, USA; c Departments of Chemistry and Biochemistry, University of Colorado at Boulder, Boulder, CO 80309, USA; d School of Materials and Metallurgy, Inner Mongolia University of Science and Technology, PR China

**Keywords:** Tom1,, GAT domain,, Tollip,, Ubiquitin,, nuclear magnetic resonance

## Abstract

Cellular homeostasis requires correct delivery of cell-surface receptor proteins (cargo) to their target subcellular compartments. The adapter proteins Tom1 and Tollip are involved in sorting of ubiquitinated cargo in endosomal compartments. Recruitment of Tom1 to the endosomal compartments is mediated by its GAT domain’s association to Tollip’s Tom1-binding domain (TBD). In this data article, we report the solution NMR-derived structure of the Tom1 GAT domain. The estimated protein structure exhibits a bundle of three helical elements. We compare the Tom1 GAT structure with those structures corresponding to the Tollip TBD- and ubiquitin-bound states.

## 1 **Specifications table**

**Table:** 

Subject area	*Biology*
More specific subject area	*Structural biology*
Type of data	*Table, text file, graph, figures*
How data was acquired	*Circular dichroism and NMR. NMR data was recorded using a Bruker 800 MHz*
Data format	*PDB format text file. Analyzed by CS-Rosetta, Protein Structure Validation Server (PSVS), NMRPipe, NMRDraw, and PyMol*
Experimental factors	*Recombinant human Tom1 GAT domain was purified to homogeneity before use*
Experimental features	*Solution structure of Tom1 GAT was determined from NMR chemical shift data*
Data source location	*Virginia and Colorado, United States.*
Data accessibility	*Data is available within this article. Tom1 GAT structural data is publicly available in the RCSB Protein Data Bank (http://www.rscb.org/) under the accession number PDB: 2n9d*

## 2 **Value of the data**

• The Tom1 GAT domain solution structure will provide additional tools for modulating its biological function.
• Tom1 GAT can adopt distinct conformations upon ligand binding.
• A conformational response of the Tom1 GAT domain upon Tollip TBD binding can serve as an example to explain mutually exclusive ligand binding events.

## 3 Data

**Fig. 1:**
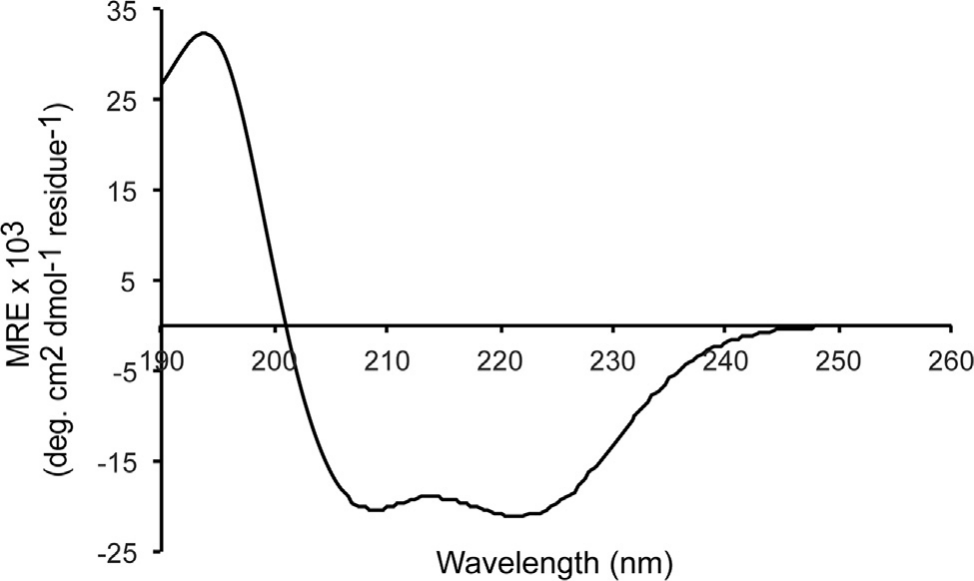
Representative far-UV CD spectrum of the His-Tom1 GAT domain.

**Fig. 2:**
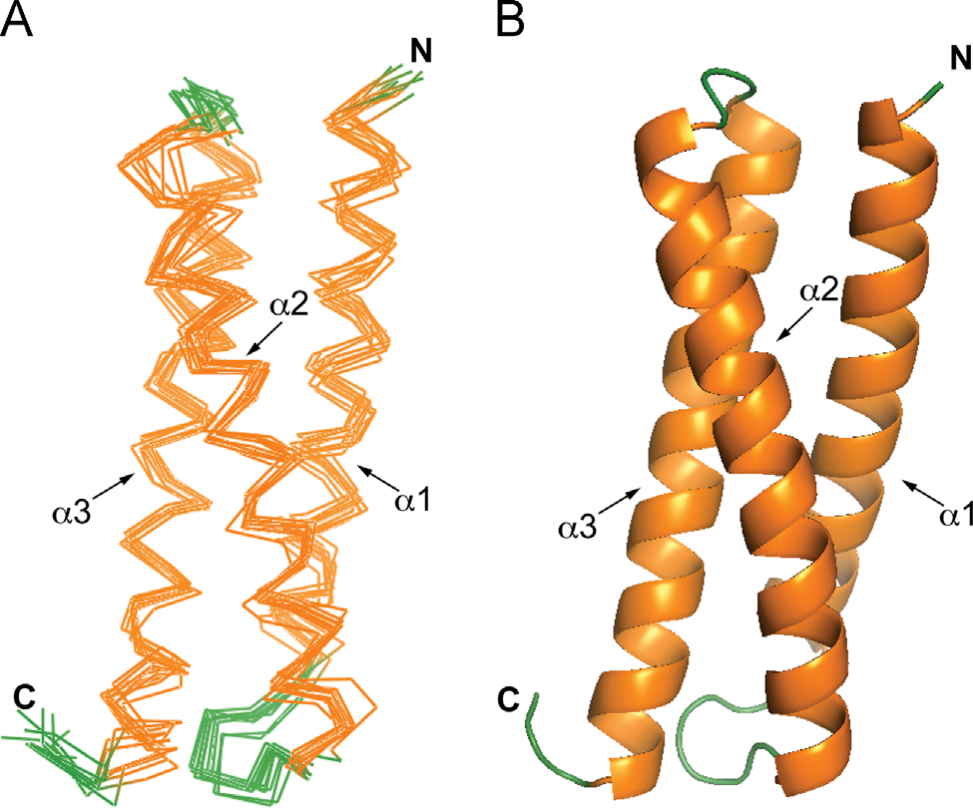
(A) Stereo view displaying the best-fit backbone superposition of the refined structures for the Tom1 GAT domain. Helices are shown in orange, whereas loops are colored in green. (B) Ribbon illustration of the Tom1 GAT domain.

**Fig. 3:**
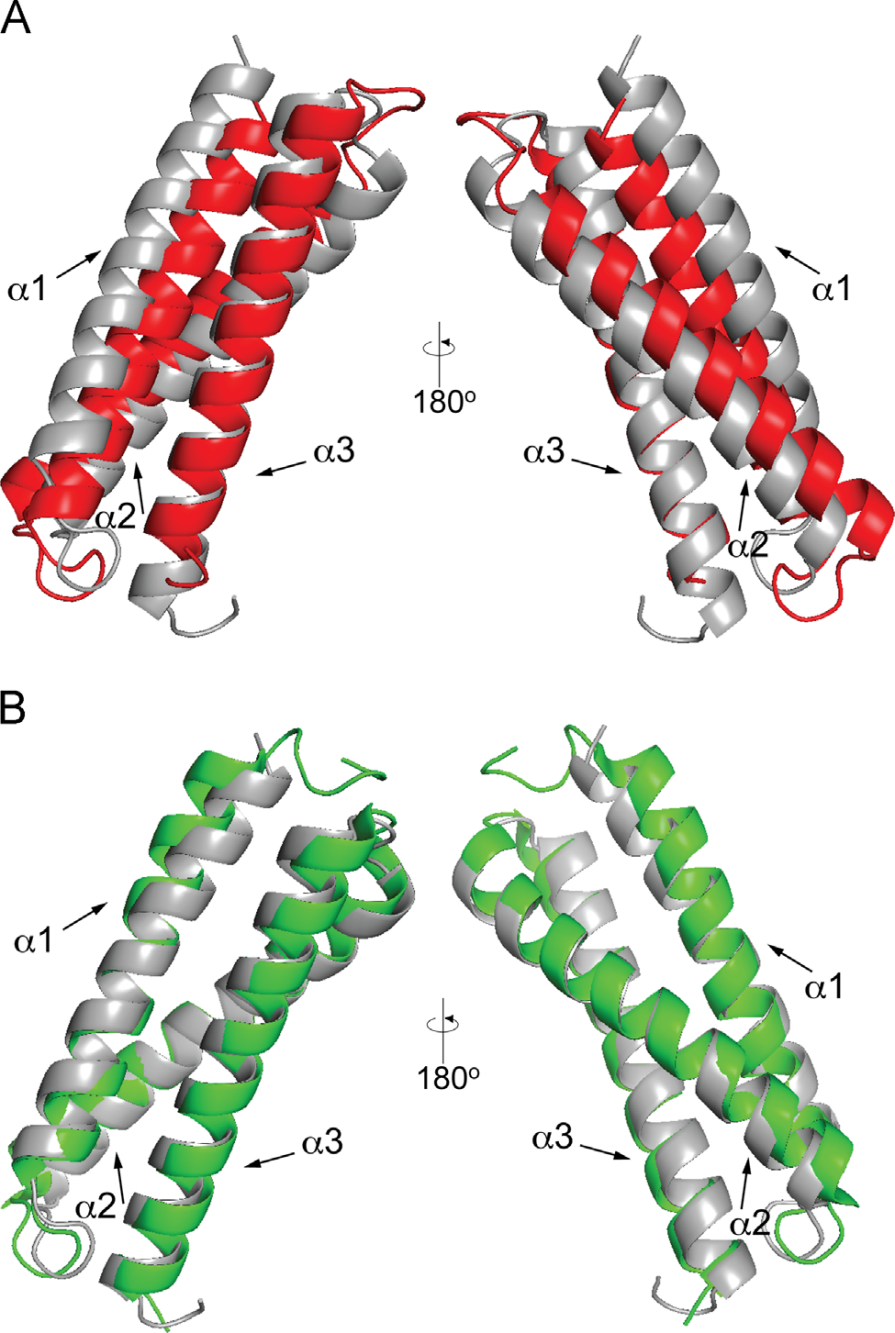
(A) Two views of the superimposed structures of the Tom1 GAT domain in the free state (gray) with that in the Tollip TBD-bound state (red). (B) Two views of the superimposed structures of the Tom1 GAT domain (gray) with that in the Ub-bound state (green).

Analysis of the far-UV circular dichroism (CD) spectrum of the Tom 1 GAT domain (Fig. 1) predicts 58.7% α-helix, 3% β-strand, 15.5% turn, and 22.8% disordered regions. The Tom1 GAT structural restraints yielded ten helical structures (Fig. 2A,B) with a root mean square deviation (RMSD) of 0.9 Å for backbone and 1.3 Å for all heavy atoms (Table 1) and estimated the presence of three helices spanning residues Q216-E240 (α-helix 1), P248-Q274 (α-helix 2), and E278-T306 (α-helix 3). Unlike ubiquitin binding, data suggest that conformational changes of the Tom1 GAT α-helices 1 and 2 occur upon Tollip TBD binding (Fig. 3A,B).

## 4 Experimental design, materials, and methods

### 4.1 Protein expression and purification

Human Tom1 GAT (residues 215–309) cDNA was cloned into both pGEX6P1 and pET28a vectors, and expressed as GST-tagged and His-tagged fusion proteins, respectively, using *Escherichia coli [Rosetta (DE3) strain]*. The ^13^C, ^15^N-labeled Tom1 GAT domain was expressed and purified as described previously [1].

### 4.2 Circular dichroism

Far-UV CD spectra of the His-Tom1 GAT domain were collected on a Jasco J-815 spectropolarimeter using a 1 mm path length quartz cell at room temperature. The protein (10 μM) was solubilized in 5 mM Tris–HCl (pH 7) and 100 mM KF. Spectra were obtained from five accumulated scans from 190 to 260 nm using a bandwidth of 1 nm and a response time of 1 s at a scan speed of 20 nm/min. Buffer backgrounds were employed to subtract the protein spectra. Data was processed using the Dichroweb server and the CONTIN algorithm (http://dichroweb.cryst.bbk.ac.uk/html/home.shtml).

### 4.3 NMR structure determination

NMR experiments were performed using 1 mM ^13^C, ^15^N-labeled Tom1 GAT domain in a buffer containing 20 mM *d*_*11*_-TrisHCl (pH 7), 50 mM KCl, 1 mM *d*_*18*_-DTT, and 1 mM NaN_3_. NMR spectra were recorded at 25 °C on a Bruker 800-MHz spectrometer (University of Virginia). The individual structure of Tom1 GAT was generated using CS-Rosetta (https://csrosetta.bmrb.wisc.edu/csrosetta). Chemical shift information (BMRB #26574) was used to obtain the structure calculation. The Rosetta calculations yielded 3000 structures of Tom1 GAT. From these, ten structures were selected based on their score and RMSDs, and converted to Protein Data Bank (PDB) format. NMR structural statistics for the ten lowest energy conformers of Tom1 GAT was generated using the Protein Structure Validation Suite. By using MolProbity, the Ramachandran analysis of the ten superimposed Tom1 GAT structures identified that 100% of the residues were in the most favored regions and there were no Ramachandran outliers in the allowed and disallowed regions. Protein structure images were obtained using PyMol (http://www.pymol.org). The structures of the ubiquitin- and Tollip TBD-bound states of the Tom1 GAT domain were obtained from data reported in Refs. [1] and [2].

## Figures and Tables

**Table 1:** NMR and refinement statistics for the Tom1 GAT domain. NMR structural statistics for lowest energy conformers of Tom1 GAT using PSVS.

	**Tom1 GAT**
**NMR distance and dihedral constraints**	
Dihedral angle restraints total	178
* ϕ*	89
* ψ*	89
**Structure statistics**	
Dihedral angle constraints (deg)	8.8±0.2
Max. dihedral angle violation (deg)	111±3
Deviations from idealized geometry	
Bond lengths (Å)	0.011
Bond angles (deg)	0.7
Average pairwise r.m.s. deviation (Å)^a^	
Protein	
Heavy	1.3
Backbone	0.9

a Pairwise backbone and heavy-atom r.m.s. deviations were obtained by superimposing residues 215–309 of Tom1 GAT among 10 lowest energy refined structures.
